# Knowledge toward COVID‐19 in children among undergraduate students at the beginning of COVID‐19 era

**DOI:** 10.1002/nop2.1600

**Published:** 2023-01-10

**Authors:** Sawsan Abuhammad, Hossam Alhawatmeh, Ahlam Al‐Natour, Shaher Hamaideh, Nasr Alrabadi, Amer Sindiani, Amat Alkhaleq Mehrass

**Affiliations:** ^1^ Faculty of Nursing Jordan University of Science and Technology Irbid Jordan; ^2^ Department of Community and Mental Health Nursing, Faculty of Nursing The Hashemite University Zarqa Jordan; ^3^ Faculty of Medicine Jordan University of Science and Technology Irbid Jordan; ^4^ Faculty of Medicine Thamar University Thamar City Yemen

**Keywords:** COVID‐19, Jordan, knowledge, undergraduate students

## Abstract

**Aims:**

To describe the level of knowledge of undergraduate students in Jordan toward COVID‐19 in children in respect of the clinical signs of the disease, modes of transmission, protection measures against the disease and satisfaction with governmental measures.

**Design:**

A cross‐section was utilized in this study.

**Methods:**

An online survey questionnaire was utilized in this research study. All undergraduate students in Jordan were able to take part. The size of the sample was 799. Knowledge toward COVID‐19 among children was used to assess the participants' knowledge about COVID‐19.

**Results:**

The findings indicate that the students had a good understanding of the clinical signs, mode of transmission and protection measures and were satisfied with governmental measures. According to the students' responses, the resource they used the most was social media followed by news channels. Our study also found that medical specialty students had more knowledge toward COVID‐19 than non‐medical.

## 1 INTRODUCTION

Little information is available regarding COVID‐19 in children. Therefore, this paper focuses on COVID‐19 in children for the following reasons: First, even though there is a low probability of children experiencing severe illness due to contracting COVID‐19 as compared to older adults, various subpopulations of children have an elevated possibility of developing a more severe form of the disease (Dong et al., 2020). Researchers monitoring viruses in a paediatric intensive care unit in China found there were more cases of coronavirus as compared to human metapneumovirus in children with acute respiratory distress syndrome (Li et al., 2019).

Moreover, in another study of admitted children in Norway, researchers discovered coronavirus in 10% of children suffering respiratory tract infections (Heimdal et al., 2019). Also, younger age, underlying pulmonary diseases, and conditions compromising the immune system have been linked with extreme results in non‐COVID‐19 infections in minors (Ogimi et al., 2019; Wei et al., 2020). Secondly, it is challenging to discern the dangers of severe ailment that may be attributable to the presence of COVID‐19 in children. Past research has shown that children who have coronavirus in their respiratory system can experience viral coinfections in approximately two‐thirds of cases (Wei et al., 2020). Moreover, according to Dong et al. (2020), the presence of other viruses is generally not analysed in a conventional manner; there is a clinical diagnosis in two‐thirds of the cases, without virology confirmation. Besides, it has also been found that there is a greater likelihood of children without virological symptoms being ill as compared to those with detected COVID‐19, probably because other pathogens have caused their symptoms (Ogimi et al., 2019; Wei et al., 2020). Third, children may play a significant role in the transmission of viruses within the community (Cruz & Zeichner, 2020). Jordan is no exception, there is an increase in coronavirus cases worldwide and Jordan is no exception, where the virus is transmitted through contact between individuals (Abuhammad, Khabour & Alzoubi., 2020).

In Jordan, the current batch of university students will soon serve society in various professional capacities. Therefore, understanding the knowledge of undergraduate students about COVID‐19 in children will allow their tutors to prepare them to manage the increasing incidence of COVID‐19. Thus, it is essential to understand the currents among these future parents and providers of healthcare and other jobs in the community. Indeed, changing the viewpoint of people around the world is a crucial goal of the World Health Organization (WHO) to lower the incidence of COVID‐19 in the general population and particularly in children. Thus, it is essential to review the knowledge toward COVID‐19 among first, second, third, fourth, fifth and sixth year undergraduate students enrolled in Bachelor of Science (B.Sc.) programs at Jordanian private and public universities. Due to this gap in the literature, the researcher performed this study. Specifically, this study aimed at (a) describing the level of knowledge of undergraduate students in Jordan toward COVID‐19 in children in respect of the clinical signs of the disease, modes of transmission, protection measures against the disease and satisfaction with governmental measures.

## 2 METHOD

### 2.1 Design

A cross‐sectional design was employed to collect data from the participants.

### 2.2 Sample and setting

All undergraduate students from all government and private universities in Jordan were eligible to participate in the study so long as they met the following inclusion criteria: (1) able to read and write in the Arabic language and (2) aged more than 18 years old. Students were excluded if they are not able to write and read the Arabic language. The quota sampling strategy used was based on the participant's age and gender to ensure that the sample was a broad representation of the general population in Jordan. The sample size of the study was calculated using G‐Power 3.1., Universitat Kiel, Germany (RRID:SCR_013726), based on convenience/quota sample method, small effect size, alpha of 0.05 and power of 0.95. The required minimum number of subjects was 750 More than 1000 questionnaires were sent out electronically to undergraduate students from all universities in Jordan. The authors used STROBE checklist to ensure meeting all the requirements for a cross‐sectional study (Ghaferi et al., 2021).

### 2.3 Measures

The following demographic characteristics were obtained through the self‐reported online survey: age, gender, living conditions, income, nationality, specialization, level of education and education of parents. The second part of the survey (Knowledge toward COVID‐19 among children) was used to assess the participants' knowledge about COVID‐19. This instrument was adopted from another study that used the instrument to measure the knowledge of parents about COVID‐19 in children (Abuhammad, 2021). The components of this instrument are the signs and symptoms of the disease (nine signs), modes of transmission (three questions), protection measures against the disease (11 statements) and satisfaction with government decisions for children (three questions). However, it should be noted that there is yet no standard measure of student knowledge about COVID‐19 in children. The Cronbach alpha for the instrument was .89. The permission to use the instrument was approved by the original authors.

### 2.4 Data collection

The researcher utilized an online questionnaire to obtain the data for this research study. Before interacting with human subjects, the researcher obtained permission from the Institutional Review Board (IRB) of Jordan University of Science and Technology (JUST) to conduct the study. Following receipt of IRB permission, the participants were recruited via online advertisement distributed on social media such as Facebook and Twitter. Anyone interested contacted the researchers to let them know they wanted to participate. The researcher sent them the consent form and the survey online via social media such as Facebook, Messenger, WhatsApp and other social media applications for people who sent that they met the inclusion criteria. To avoid bias, the link of the study was mentioned in the cover letter of the electronic survey that people who did not meet the inclusion criteria, and they should not participate in the study. More than 1000 questionnaires were sent to undergraduate students in public and private universities in Jordan.

### 2.5 Ethical consideration

Jordan University of Science and Technology IRB approved this study. The researcher approached eligible participants and issued them with detailed information regarding the objective of the study. This information included the following statements: “The researcher will utilize the information in assisting the community and the university; the researcher will keep the student information private; the researcher will not use any information in presentations or publications in a manner that will result in your [the student's] exposure to the Principal Investigator and the Office for Human Research Protection at (243/2020)”.

### 2.6 Data analysis

The Statistical Package for the Social Sciences version 25 was used for all statistical analyses (IBM, Armonk, NY, USA). The results of the descriptive and bivariate analyses were deemed significant based on an alpha level of less than .05. The Kolmogorov–Smirnov test was used to evaluate the normality of all continuous variables. Any discrepant variables were transformed into more distributed scores. The subjects' responses were evaluated for each variable (measure). If each variable had 40% or more missing data, then the participants' responses for that measure were deleted list‐wise (10). Where the amount of missing data was below 40% this imputed from the mean of the responses to the items for each variable. Descriptive statistics were used to describe the research subjects' demographics and the total scores for each of the variables. Outliers were determined and eliminated prior to the data analysis.

## 3 RESULTS

### 3.1 Demographic data

A total of 1000 questionnaires were distributed, and 799 undergraduates filled in and returned the questionnaire. Therefore, the response rate was 79.9%. Out of the 799 participants, 528 were female (66.3%) and 263 were male (37.3%). The mean age of the participants was 21 years. See Table 1 for more information.

**TABLE 1:** Demographical characteristics of the participants (*n* = 799)

		Frequency	Percent
Gender	Male	194	24.3
	Female	604	75.7
Religion	Islam	782	98.0
	Others	16	2.0
Income	Less Than 400	364	45.6
	400 To 600	184	23.1
	600 To 800	98	12.3
	More Than 800	74	9.3
	5	78	9.8
Living	City	466	58.4
	Village	332	41.6
Nationality	Jordanian	756	94.7
	Other	42	5.3
Medical Vs Non‐Medical	Medical	395	49.4
	Non‐Medical	404	50.6
Edu	First Year	128	16.0
	Second Year	188	23.6
	Third Year	138	17.3
	Fourth Year	176	22.1
	Fifth Year	12	1.5
	Sixth Year	156	19.5
Father Education	Secondary	254	31.8
	Associate	124	15.5
	Bachelor	232	29.1
	Graduate	188	23.6
Mother Education	Secondary	206	25.8
	Associate	124	15.5
	Bachelor	270	33.8
	Graduate	198	24.8

### 3.2 Sources of information

There are many resources from which to gain information regarding COVID‐19. According to the students' responses, the resource they used the most was social media (*n* = 520, 65%) followed by news channels (*n* = 532, 66.5%). The least used source was other sources such as newspapers (*n* = 50, 6.2%). See Table 2.

**TABLE 2:** Sources of information on COVID‐19 in children

Source	Number	Percentage
Social media	520	65.0%
Google and search engines	238	29.7%
Family, friends, neighbours	168	21.0%
News channels	532	66.5%
Ministry of Health website	508	63.5%
Scientific articles and research	300	37.5%
Other, such as newspapers	50	06.2%

### 3.3 Clinical signs and modes of transmission

Information was collected regarding the students' knowledge about the signs and symptoms of COVID‐19 in children. Many correctly stated that fever (*n* = 720, 90.1%) was a clinical sign, followed by cough (*n* = 582, 72.8%). Only a few reported that red eyes (*n* = 102, 12.7%) constituted a clinical sign. As regards transmission, the three main modes that were recognized by the students were touching surfaces such as door handles and tables (*n* = 700, 87.6%), coughing and sneezing (*n* = 686, 85.8%), and shaking hands (604, 75.5%). See Table 3.

**TABLE 3:** Signs and symptoms of COVID‐19 in children according to students' responses

Clinical signs	Number	Percentage
Fever	720	90.1%
Cough	582	72.8%
Runny nose	212	26.5%
Sore throat	492	61.5%
Shortness of breath	440	55.0%
Joint/muscle pain	172	21.5%
Red eyes	102	12.7%
Skin rash	148	18.5%
Diarrhoea	180	22.5%
Vomiting	176	22.0%
Asymptomatic	380	47.5%

### 3.4 Measures to protect against COVID‐19 in children

Many measures can be taken to prevent the spread of COVID‐19. Students were asked to choose from multiple options for possible ways to prevent COVID‐19 in children. Almost all the students answered that “Quarantine for all family members (stay at home) to protect children” (*n* = 792, 99.9%) and that “Educating people about coronavirus in children is important to prevent the spread of the disease” (*n* = 780, 97.7%). However, very few students answered “know how to apply social separation with family children” as an effective way to reduce the incidence of the disease (*n* = 22, 2.8%). See Table 4. As regards the students' opinions regarding government measures to protect against COVID‐19 in children, the responses showed that many students supported the government in respect of the measures it was taking against COVID‐19 (*n* = 644, 87%). Also, most students supported the government measures that required that children should stay at home all the time (*n* = 792, 99.2%) and that schools suspend their operations (*n* = 788, 98.7%).

**TABLE 4:** Undergraduate students' knowledge on measures to protect against COVID‐19 in children

Measure	Number	Percentage
Clean hands frequently by rubbing hands with hand sanitizer or water and soap	746	93.3%
Eat boiled and cooked food	378	47.3%
Apply mask to known or suspected patients	596	74.6%
Place known or suspected patients in well‐ventilated areas: individual rooms	640	80.1%
All health workers should wear protective clothing	610	76.3%
Avoid transporting patients and transporting them outside their area of residence unless necessary	560	70.1%
Routinely clean and disinfect surfaces in contact with known or suspected patients	642	80.3%
Quarantine all family members (stay at home) to protect them	792	99.9%
Educating people about coronavirus in children is important to prevent the spread of the disease	780	97.7%
Apply social separation with family children	22	2.8%
Give coronavirus vaccinations to children when available	780	97.7%
When any child in the family feels sick, seek medical attention immediately	492	61.7%
Pet animals may transmit coronavirus to children and should be avoided	746	93.5%
Individuals should avoid taking children to crowded places and avoid using public transportation	704	88.2%
Educating people about coronavirus in children is important to prevent the spread of the disease	780	97.7%

## 4 DISCUSSION

This study aimed to describe the level of knowledge of undergraduate students in Jordan toward COVID‐19 in children in respect of the clinical signs of the disease, modes of transmission, protection measures against the disease and satisfaction with governmental measures. Our results showed that the undergraduate students in this study depended mainly on social media and news channels for their information on COVID‐19. This finding is consistent with Depoux et al. (2020), who found that people rely heavily on social media channels such as Facebook and Twitter for the latest news.

Our results also showed that the undergraduate students had adequate knowledge about the clinical signs of COVID‐19 that include fever, exhaustion, production of sputum, breathlessness, cough, headache and sore throat. This is congruent with the literature, which indicates that the most frequent clinical signs are fever, exhaustion, production of sputum, breathlessness, cough, headache and sore throat (Chen et al., 2020). Besides, there can be a manifestation of gastrointestinal symptoms in some patients, such as diarrhoea and vomiting. Three studies were conducted in 41 and 138 children's patient investigations in Hubei province (Chen et al., 2020; Huang et al., 2020). In the first study of them, fever and cough were found to be the dominant manifestations and rarely upper respiratory and gastrointestinal signs, which indicates that there may be the differences in viral tropism in comparison to other previous respiratory outbreaks such as influenza (Wang et al., 2016).

One probable reason children seem less prone to contracting COVID‐19 in the beginning of the outbreak is that they perform minimal outdoor activities and undertake minimal travel to international destinations, thus lowering their probability of getting the virus. However, there may still be a future increase in the number of paediatric patients. It remains a mystery why children are relatively resistant to some infectious ailments (Sinha et al., 2020). However, it has been suggested that developmental changes in the axonal transport system may give the answer to the greater immunity in baby mice to paralysis triggered by the polio vaccine. Some other possible reasons may be the innate immune system in children has a continually active response and that children have a healthier respiratory system because they have not been in as much contact with pollutants as compared to older individuals and have minimal hidden complications due to their early age (Sinha et al., 2020). However, a more robust immune system in adults may explain the disadvantageous immunity that is linked to acute respiratory distress syndrome. Differences in the distribution, development and functioning of virus receptors are often linked with the level of incidence that is associated with differences in age (Ludvigsson, 2020; Sinha et al., 2020).

Our study found that many undergraduate students think that COVID‐19 affects children to the same degree as adults and many think coronavirus poses a threat to children's lives. Previous research has shown that there is a more common adult COVID‐19 infection compared with children (Lee et al., 2019; Lu et al., 2020), this outcome indicates that children may indeed be quite resistant to SARS‐CoV‐2. It has been determined that the expression of ACE2 in the lungs of rats significantly reduces with age. This result may be different from the relative susceptibility of children to COVID‐19. Therefore, it is essential to discuss the principal factors that may assist in caring for child patients who have COVID‐19 infection (Thompson & Rasmussen, 2020).

As for modes of transmission, our study found that most of the undergraduate students recognized very well the modes of transmission of COVID‐19. Many students reported that sources of transmission include touching surfaces such as door handles and tables, coughing and sneezing, and shaking hands. Currently, research and development of anti‐virus immunizations is underway in China. At the time of writing, it seems that COVID‐19 moves from individual to individual through similar methods as the common cold and flu, i.e. encountering sneezing or coughing aerosols or encountering fluids from infected persons (Shen et al., 2020; Sinha et al., 2020). The function of faecal‐oral transmission has not yet been determined in COVID‐19. However, it was found to occur during the SARS outbreak (Sinha et al., 2020).

In our study, many students reported that “Quarantine for all family members (stay at home) to protect children” and “Educating people about COVID‐19 in children is important to prevent the spread of the disease.” On the contrary, only a few students answered that “Applying social separation with family children” is an effective way to combat the disease. Methods of lowering COVID‐19 transmission include personal and environmental actions, determining and separating incidences, tracing contacts and quarantining, implementing measures on social and physical distancing including the banning of mass gatherings, setting rules for international travel (Adalja et al., 2020; Chinazzi et al., 2020), providing relevant treatments and conducting vaccinations.

In our study, many students supported the government measure that requires that children should stay at home all the time to tackle the coronavirus epidemic. The WHO (2020) has provided many steps to ensure the safety and prevent COVID‐19 in children. These steps include the physical separation of individuals (at least 1 m) and reduced contact in infected areas as well as promoting and maintaining virtual contact with relatives and communities (Abuhammad, 2020; Anderson et al., 2020). There are also general measures that can be taken to ensure the safety of everyone, especially children. These include implementing flexible methods of working such as teleworking, offering distance learning to students, minimizing and preventing gatherings, as well as closing non‐essential facilities and services (Abuhammad et al., 2020; Adalja et al., 2020; Muflih et al., 2021), protecting and securing vulnerable populations, restricting the local or national movements of individuals, implementing or enforcing measures to stay at home (Cauchemez et al., 2009) and reorganizing healthcare and social services to secure and support hospitals (Anderson et al., 2020; Keeling et al., 2020). These measures are enacted together with personal protective actions against COVID‐19 such as frequent washing of hands and good hygiene manners when coughing (Chinazzi et al., 2020; Fisher & Heymann, 2020; Thompson & Rasmussen, 2020).

Our findings contradict the results of previous literature, an assumption can be made that nonpharmacological strategies are useful from an individual perspective, for instance, fluid support, oxygen and ventilation support (Ali & Gatiti, 2020; Wu & McGoogan., 2020). Non‐pharmaceutical measures remain important in managing COVID‐19 because of the absence of official vaccines or antivirals for coronavirus (Abuhammad et al., 2022; Shen et al., 2020).

### 4.1 Strengths and limitations

One of the strengths of this study is using many students and recruiting them over social media to reach the highest number of students. The limitations of the study include using a cross‐sectional design, which limits the cause‐and‐effect relationship. Another limitation is using this study in Jordan, which limits the generalizability to other countries.

## 5 CONCLUSION

In summary, the students showed satisfactory‐level knowledge of COVID‐19 in children. The students had knowledge about the signs and symptoms of disease, ways of transmission, protection measures and satisfaction with measures to combat COVID‐19 in children, considering the importance of possessing knowledge about this ongoing global public health emergency.

## AUTHOR CONTRIBUTIONS

All authors participated in all steps of this study.

## FUNDING INFORMATION

This research did not receive a grant.

## CONFLICT OF INTEREST

The authors have no conflict of interest to disclose.

## Data Availability

Data will be avaliable upon request.

## References

[nop21600-bib-0002] Abuhammad, S. (2020). Barriers to distance learning during the COVID‐19 outbreak: A qualitative review from parents' perspective. Heliyon, 6(11), e05482. 10.1016/j.heliyon.2020.e05482 33200106 PMC7654229

[nop21600-bib-0003] Abuhammad, S. (2021). Parents' knowledge and towards COVID‐19 in children: A Jordanian study. International Journal of Clinical Practice, 75(2), e13671. 10.1111/ijcp.13671 32780560 PMC7435566

[nop21600-bib-0004] Abuhammad, S. , Alzoubi, K. H. , & Khabour, O. (2021). Fear of COVID‐19 and stigmatization towards infected people among Jordanian people. International Journal of Clinical Practice, 75(4), e13899. 10.1111/ijcp.13899 33280204 PMC7883188

[nop21600-bib-0005] Abuhammad, S. , Khabour, O. F. , & Alzoubi, K. H. (2020). COVID‐19 contact‐tracing technology: Acceptability and ethical issues of use. Patient Preference and Adherence, 14 , 1639 – 1647. 10.2147/PPA.S276183 32982188 PMC7509307

[nop21600-bib-0006] Abuhammad, S. , Khabour, O. F. , Alzoubi, K. H. , El‐zubi, F. , & Hamaideh, S. H. (2022). Respiratory infectious diseases and adherence to nonpharmacological interventions for overcoming COVID‐19 pandemic: A self‐reported study. International Journal of Clinical Practice, 2022 , 1 – 6. 10.1155/2022/4495806 PMC9159161 35685528

[nop21600-bib-0007] Adalja, A. A. , Toner, E. , & Inglesby, T. V. (2020). Priorities for the US health community responding to COVID‐19. JAMA, 6(4), 45 – 53. 10.1001/jama.2020.3413 32125355

[nop21600-bib-0008] Ali, M. Y. , & Gatiti, P. (2020). The COVID‐19 (coronavirus) pandemic: Reflections on the roles of librarians and information professionals. Health Information and Libraries Journal, 37 , 158 – 162. 10.1111/hir.12307 32251543

[nop21600-bib-0009] Anderson, R. M. , Heesterbeek, H. , Klinkenberg, D. , & Hollingsworth, T. D. (2020). How will country‐based mitigation measures influence the course of the COVID‐19 epidemic? The Lancet, 395(10228), 931 – 934. 10.1016/S0140-6736(20)30567-5 PMC7158572 32164834

[nop21600-bib-0010] Cauchemez, S. , Ferguson, N. M. , Wachtel, C. , Tegnell, A. , Saour, G. , Duncan, B. , & Nicoll, A. (2009). Closure of schools during an influenza pandemic. The Lancet Infectious Diseases, 9(8), 473 – 481. 19628172 10.1016/S1473-3099(09)70176-8 PMC7106429

[nop21600-bib-0011] Chen, N. , Zhou, M. , Dong, X. , Qu, J. , Gong, F. , Han, Y. , Qiu, Y. , Wang, J. , Liu, Y. , Wei, Y. , Xia, J. , Yu, T. , Zhang, X. , & Zhang, L. (2020). Epidemiological and clinical characteristics of 99 cases of 2019 novel coronavirus pneumonia in Wuhan, China: A descriptive study. The Lancet, 395(10223), 507 – 513. 10.1016/S0140-6736(20)30211-7 PMC7135076 32007143

[nop21600-bib-0012] Chinazzi, M. , Davis, J. T. , Ajelli, M. , Gioannini, C. , Litvinova, M. , Merler, S. , Pastore Y Piontti, A. , Mu, K. , Rossi, L. , Sun, K. , Viboud, C. , Xiong, X. , Yu, H. , Halloran, M. E. , Longini, I. M. , Jr. , & Vespignani, A. (2020). The effect of travel restrictions on the spread of the 2019 novel coronavirus (COVID‐19) outbreak. Science, 368 , 395 – 400. 10.1126/science.aba9757 32144116 PMC7164386

[nop21600-bib-0013] Cruz, A. T. , & Zeichner, S. L. (2020). COVID‐19 in children: Initial characterization of the pediatric disease. Pediatrics, 145(6), e20200834 . 10.1542/peds.2020-0834 32179659

[nop21600-bib-0014] Depoux, A. , Martin, S. , Karafillakis, E. , Preet, R. , Wilder‐Smith, A. , & Larson, H. (2020). The pandemic of social media panic travels faster than the COVID‐19 outbreaks. Journal of Travel Medicine, 27(3), taaa031 . 10.1093/jtm/taaa031 32125413 PMC7107516

[nop21600-bib-0015] Dong, Y. , Mo, X. , Hu, Y. , Qi, X. , Jiang, F. , Jiang, Z. , & Tong, S. (2020). Epidemiology of COVID‐19 among children in China. Pediatrics, 145 , e20200702 . 10.1542/peds.2020-0702 32179660

[nop21600-bib-0016] Fisher, D. , & Heymann, D. (2020). Q&A: The novel coronavirus outbreak causing COVID19. BMC Medicine, 18(1), 1 – 3. 10.1186/s12916-020-01533-w 32106852 PMC7047369

[nop21600-bib-0017] Ghaferi, A. A. , Schwartz, T. A. , & Pawlik, T. M. (2021). STROBE reporting guidelines for observational studies. JAMA Surgery, 156(6), 577 – 578. 10.1001/jamasurg.2021.0528 33825815

[nop21600-bib-0018] Heimdal, I. , Moe, N. , Krokstad, S. , Christensen, A. , Skanke, L. H. , Nordbø, S. A. , & Døllner, H. (2019). Human coronavirus in hospitalized children with respiratory tract infections: A 9‐year population‐based study from Norway. The Journal of Infectious Diseases, 219(8), 1198 – 1206. 10.1093/infdis/jiy646 30418633 PMC7107437

[nop21600-bib-0019] Huang, C. , Wang, Y. , Li, X. , Ren, L. , Zhao, J. , Hu, Y. , Zhang, L. , Fan, G. , Xu, J. , Gu, X. , Cheng, Z. , Yu, T. , Xia, J. , Wei, Y. , Wu, W. , Xie, X. , Yin, W. , Li, H. , Liu, M. , … Cao, B. (2020). Clinical features of patients infected with 2019 novel coronavirus in Wuhan, China. The Lancet, 395(10223), 497 – 506. 10.1016/S0140-6736(20)30183-5 PMC7159299 31986264

[nop21600-bib-0020] Keeling, M. J. , Hollingsworth, T. D. , & Read, J. M. (2020). The efficacy of contact tracing for the containment of the 2019 Novel coronavirus (COVID‐19). Journal of Epidemiology and Community Health, 74(10), 861 – 866. 10.1136/jech-2020-214051 32576605 PMC7307459

[nop21600-bib-0021] Lee, K. H. , Yoo, S. G. , Cho, Y. , Kwon, D. E. , la, Y. , Han, S. H. , Kim, M. S. , Choi, J. S. , Kim, S. I. , Kim, Y. S. , Min, Y. H. , Cheong, J. W. , Kim, J. S. , & Song, Y. G. (2019). Characteristics of community‐acquired respiratory viruses' infections except seasonal influenza in transplant recipients and non‐transplant critically ill patients. Journal of Microbiology, Immunology, and Infection, 12(34), 34–39. 10.1016/j.jmii.2019.05.007 PMC7102620 31262511

[nop21600-bib-0022] Li, Y. T. , Liang, Y. , Ling, Y. S. , Duan, M. Q. , Pan, L. , & Chen, Z. G. (2019). The spectrum of viral pathogens in children with severe acute lower respiratory tract infection: A 3‐year 18 prospective study in the pediatric intensive care unit. Journal of Medical Virology, 91(9), 1633–1642. 10.1002/jmv.25502 31081548 PMC7167151

[nop21600-bib-0023] Lu, R. , Zhao, X. , Li, J. , Niu, P. , Yang, B. , Wu, H. , Wang, W. , Song, H. , Huang, B. , Zhu, N. , Bi, Y. , Ma, X. , Zhan, F. , Wang, L. , Hu, T. , Zhou, H. , Hu, Z. , Zhou, W. , Zhao, L. , … Tan, W. (2020). Genomic characterisation and epidemiology of 2019 novel coronavirus: Implications for virus origins and receptor binding. The Lancet, 395(10224), 565–574. 10.1016/S0140-6736(20)30251-8 PMC7159086 32007145

[nop21600-bib-0024] Ludvigsson, J. F. (2020). Systematic review of COVID‐19 in children show milder cases and a better prognosis than adults. Acta Paediatrica, 14(3), 34–38. 10.1111/apa.15270 PMC7228328 32202343

[nop21600-bib-0025] Muflih, S. M. , Al‐Azzam, S. , Abuhammad, S. , Jaradat, S. K. , Karasneh, R. , & Shawaqfeh, M. S. (2021). Pharmacists' experience, competence and perception of telepharmacy technology in response to COVID‐19. International Journal of Clinical Practice, 75(7), e14209. 10.1111/ijcp.14209 33819372 PMC8250242

[nop21600-bib-0026] Ogimi, C. , Englund, J. A. , Bradford, M. C. , Qin, X. , Boeckh, M. , & Waghmare, A. (2019). Characteristics and outcomes of coronavirus infection in children: The role of viral factors and an immunocompromised state. Journal of the Pediatric Infectious Diseases Society, 8(1), 21–28. 10.1093/jpids/pix093 29447395 PMC6437838

[nop21600-bib-0027] Shen, K. , Yang, Y. , Wang, T. , Zhao, D. , Jiang, Y. , Jin, R. , Zheng, Y. , Xu, B. , Xie, Z. , Lin, L. , Shang, Y. , Lu, X. , Shu, S. , Bai, Y. , Deng, J. , Lu, M. , Ye, L. , Wang, X. , Wang, Y. , … Global Pediatric Pulmonology Alliance . (2020). Diagnosis, treatment, and prevention of 2019 novel coronavirus infection in children: experts' consensus statement. World Journal of Pediatrics, 1–9, 223–231. 10.1007/s12519-020-00343-7 PMC7090771 32034659

[nop21600-bib-0028] Sinha, I. P. , Harwood, R. , Semple, M. G. , Hawcutt, D. B. , Thursfield, R. , Narayan, O. , Kenny, S. E. , Viner, R. , Hewer, S. L. , & Southern, K. W. (2020). COVID‐19 infection in children. The Lancet Respiratory Medicine., 45(3), 56 – 65. 10.1016/S2213-2600(20)30152-1 PMC7154504 32224304

[nop21600-bib-0029] Thompson, L. A. , & Rasmussen, S. A. (2020). What does the coronavirus disease 2019 (COVID19) mean for families? JAMA Pediatrics, 19(2), 34–39. 10.1001/jamapediatrics.2020.0828 32167533

[nop21600-bib-0030] Wang, H. , Xiao, X. , Lu, J. , Chen, Z. , Li, K. , Liu, H. , Luo, L. , Wang, M. , & Yang, Z. (2016). Factors associated with clinical outcome in 25 patients with avian influenza a (H7N9) infection in Guangzhou, China. BMC Infectious Diseases, 16(1), 534. 10.1186/s12879-016-1840-4 27716101 PMC5048464

[nop21600-bib-0031] Wei, M. , Jingping Yuan, Y. , Liu, T. F. , Xue, Y. , & Zhang, Z.‐J. (2020). Novel coronavirus infection in hospitalized infants under 1 year of age in China. JAMA, 9(4), 23–29. 10.1186/s12879-016-1840-4 PMC7042807 32058570

[nop21600-bib-0032] World Health Organization . (2020). Coronavirus disease 2019 (COVID‐19): Situation report 72 . World Health Organization.

[nop21600-bib-0033] Wu, Z. , & McGoogan, J. M. (2020). Characteristics of and important lessons from the coronavirus disease 2019 (COVID‐19), 7(5), 6–11. 10.1001/jama.2020.2648 32091533

